# Editorial: Long Term User Training and Preparation to Succeed in a Closed-Loop BCI Competition

**DOI:** 10.3389/fnhum.2022.869700

**Published:** 2022-03-24

**Authors:** Gernot R. Müller-Putz, Damien Coyle, Fabien Lotte, Jing Jin, David Steyrl

**Affiliations:** ^1^Institute of Neural Engineering, Graz University of Technology, Graz, Austria; ^2^BioTechMed Graz, Graz, Austria; ^3^Intelligent Systems Research Centre, Ulster University, Derry, United Kingdom; ^4^Inria Bordeaux Sud-Ouest/LaBRI (Univ. Bordeaux, CNRS, Bordeaux INP), Talence, France; ^5^School of Information Science and Technology, East China of University of Science and Technology, Shanghai, China; ^6^Department of Cognition, Emotion, and Methods in Psychology, University of Vienna, Vienna, Austria

**Keywords:** Brain-Computer Interface, electroencephalogram, transfer learning, user learning, user-centered training, closed-loop learning

## Introduction

Brain-Computer Interfaces (BCIs) have been researched now for about 30 years. However, even still, much of the work done in the lab environment is seldom applied within the targeted end-users, for example, people with severe motor disabilities.

The main goal of the research community should be to finally bring the BCI into a state in which the end-users can profit and gain independence and quality of life. One possibility to push the field into practical applications is driven by initiatives such as CYBATHLON [initiated by ETH Zurich (Riener, 2016)] and other competitions. Such a competition challenges research institutions and industry to demonstrate their developments in real-world scenarios and push the boundaries of research.

In the BCI Race, at CYBATHLON (Novak et al., 2017), end-users are the pilots and they control an avatar in a race against other pilots, by using a multi-class BCI. This competition and others are enormously demanding to the developers, as the BCI system must work properly at the time of the competition, out of the lab in a foreign environment, with spectators around, noise and without second chances.

In China, a BCI competition was firstly organized by Tsinghua University in 2010. Since 2017, the BCI competition has been organized by China Electronics Society as part of World Robotics Conference. Thousands of users participate every year. This BCI competition consists of two parts, a user competition and an algorithm competition. Winners of the user competition compete then in the algorithm competition to test the performance of algorithms which were uploaded by BCI research teams. Through these BCI competitions, a large amount of BCI data for further research has been obtained, which has been used to promote the progress of BCI algorithms. In the near future, these data will be published online for BCI researchers all over the world.

Another extremely important factor, of course, is the preparation of the team for the competition. In particular, the end-user pilot should be trained to produce stable and accurate mental states that produce consistent brain oscillations to control the BCI, even in a potentially stressful environment such as in the CYBATHLON race arena.

## Research Topic Coverage

In this Research Topic we collected five original scientific papers from researchers and teams who have participated in such competitions, with a special focus on the transition from first contact with a BCI to the final control of the application.

Jiang et al. propose a novel feature selection approach for motor imagery based BCIs. They suggest using multiple time segments during the imagery task. Features, extracted by common spatial patterns, were combined to form a new feature vector and optimal temporal combination patterns were selected for classification and achieved significantly improved performance compared to traditional methods.

In Benaroch et al., the authors proposed a new BCI design based on adaptive Riemannian classifiers to address both inter and intra-session variabilities, which enabled significant user learning. They also identified a new type of BCI user learning, in which the user produced EEG patterns that were increasingly more similar to the EEG patterns used to train the BCI classifier.

Hehenberger et al. report on a 4-class BCI which they trained over a period of 14 month with an end user. They applied inter-session transfer learning to reduce calibration time and to reinforce stability of brain-pattern. It could also be demonstrated that this long-term training helped to significantly improve the performance, both in calibration but also in game performance.

Turi et al. demonstrate that the emotional state of the user impacts performance when learning to control a four-class mental imagery BCI and present a multi-stage, user-centered training protocol to enable successful control, even in stressful situations, such as training for a competitive BCI race.

Robinson et al. show that closed-loop BCI calibration paradigms with real-time feedback are more engaging for the pilot, achieve better online performance, and lead to more localized brain activation patterns when compared to conventional open-loop BCI calibration paradigms.

All articles in this collection showed important contributions to address EEG signal and BCI performance variabilities typically observed in practical BCI use outside the lab, over long term use. In particular, the research showed that both inter-session and intra-session variabilities should be addressed to obtain optimal performances. This can be achieved at the machine level, e.g., by using transfer learning and/or adaptive machine learning algorithms, but also at the user learning level, by considering the states of the user, the feedback provided or the context of the training (e.g., game context vs. lab training context). Ideally, both aspects should be considered together, but how to do so optimally is still an open question.

## Author Contributions

All authors listed have made a substantial, direct, and intellectual contribution to the work and approved it for publication.

## Conflict of Interest

The authors declare that the research was conducted in the absence of any commercial or financial relationships that could be construed as a potential conflict of interest.

## Publisher's Note

All claims expressed in this article are solely those of the authors and do not necessarily represent those of their affiliated organizations, or those of the publisher, the editors and the reviewers. Any product that may be evaluated in this article, or claim that may be made by its manufacturer, is not guaranteed or endorsed by the publisher.
